# Multivariate Analysis of Traumatic Brain Injury: Development of an Assessment Score

**DOI:** 10.3389/fneur.2015.00068

**Published:** 2015-03-30

**Authors:** John E. Buonora, Angela M. Yarnell, Rachel C. Lazarus, Michael Mousseau, Lawrence L. Latour, Sandro B. Rizoli, Andrew J. Baker, Shawn G. Rhind, Ramon Diaz-Arrastia, Gregory P. Mueller

**Affiliations:** ^1^Department of Anatomy, Physiology and Genetics, Uniformed Services University of the Health Sciences, Bethesda, MD, USA; ^2^U.S. Army Graduate Program in Anesthesia Nursing, Academy of Health Sciences, Joint Base San Antonio, Fort Sam Houston, TX, USA; ^3^Behavioral Biology Branch, Center for Military Psychiatry and Neuroscience Research, Walter Reed Army Institute of Research, Silver Spring, MD, USA; ^4^Stroke Branch, National Institute of Neurological Disorders and Stroke, Bethesda, MD, USA; ^5^Defence Research and Development Canada, Toronto Research Centre, Toronto, ON, Canada; ^6^Department of Anesthesia, Keenan Research Centre of the Li Ka Shing Knowledge Institute, St Michael’s Hospital, University of Toronto, Toronto, ON, Canada; ^7^Department of Surgery, Keenan Research Centre of the Li Ka Shing Knowledge Institute, St Michael’s Hospital, University of Toronto, Toronto, ON, Canada; ^8^Department of Critical Care Medicine, Keenan Research Centre of the Li Ka Shing Knowledge Institute, St Michael’s Hospital, University of Toronto, Toronto, ON, Canada; ^9^Brain Injury Laboratory, Li Ka Shing Knowledge Institute, Cara Phelan Centre for Trauma Research, Keenan Research Centre University of Toronto, Toronto, ON, Canada; ^10^Center for Neuroscience and Regenerative Medicine, Uniformed Services University of the Health Sciences, Bethesda, MD, USA

**Keywords:** biomarkers, assessment score, human, mild traumatic brain injury, multivariate analysis

## Abstract

Important challenges for the diagnosis and monitoring of mild traumatic brain injury (mTBI) include the development of plasma biomarkers for assessing neurologic injury, monitoring pathogenesis, and predicting vulnerability for the development of untoward neurologic outcomes. While several biomarker proteins have shown promise in this regard, used individually, these candidates lack adequate sensitivity and/or specificity for making a definitive diagnosis or identifying those at risk of subsequent pathology. The objective for this study was to evaluate a panel of six recognized and novel biomarker candidates for the assessment of TBI in adult patients. The biomarkers studied were selected on the basis of their relative brain-specificities and potentials to reflect distinct features of TBI mechanisms including (1) neuronal damage assessed by neuron-specific enolase (NSE) and brain derived neurotrophic factor (BDNF); (2) oxidative stress assessed by peroxiredoxin 6 (PRDX6); (3) glial damage and gliosis assessed by glial fibrillary acidic protein and S100 calcium binding protein beta (S100b); (4) immune activation assessed by monocyte chemoattractant protein 1/chemokine (C–C motif) ligand 2 (MCP1/CCL2); and (5) disruption of the intercellular adhesion apparatus assessed by intercellular adhesion protein-5 (ICAM-5). The combined fold-changes in plasma levels of PRDX6, S100b, MCP1, NSE, and BDNF resulted in the formulation of a TBI assessment score that identified mTBI with a receiver operating characteristic (ROC) area under the curve of 0.97, when compared to healthy controls. This research demonstrates that a profile of biomarker responses can be used to formulate a diagnostic score that is sensitive for the detection of mTBI. Ideally, this multivariate assessment strategy will be refined with additional biomarkers that can effectively assess the spectrum of TBI and identify those at particular risk for developing neuropathologies as consequence of a mTBI event.

## Introduction

Mild traumatic brain injury (mTBI) is a major problem for civilian and military clinicians alike and is often described as an invisible wound (1). Such an injury can precipitate a cascade of intracerebral events that include hypoxia, oxidative stress, necrosis, apoptosis, and chronic inflammation (2–4). These processes may be initiated in the absence of any acute clinical signs (5, 6). While many patients never exhibit overt clinical signs of mTBI, a subset of mTBI cases, estimated to be up to 20%, proceed to develop injury-related pathologies over time (7, 8). Accordingly, a long-sought goal for the treatment of mTBI is the identification of patients who are at risk of developing long-term complications. A missed diagnosis of TBI undermines the opportunity for immediate clinical treatments and masks the need for behavioral measures designed to prevent the occurrence of a second head injury (9). Additionally, missed diagnoses are key factors hindering preclinical and clinical TBI investigations (10, 11).

Clinical assessment for TBI routinely involves acute injury surveillance, neuropsychological testing, brain imaging, and recording of signs and symptoms (9, 12, 13). By definition, however, none of these methods are sensitive for the diagnosis of mTBI (14, 15). For more than a decade, a large effort has focused on the identification of blood-borne biomarkers that are capable of revealing the otherwise invisible injury of mTBI (16). Unfortunately, the success of these endeavors has been limited and at present, the biomarker strategy has yet to produce a diagnostic tool that is sensitive and specific for the identification of mTBI (17–21). Key factors limiting the success of these efforts have included the lack of candidates for investigation as well as the focus of many studies on the potential of just a single candidate as being informative in mTBI (20, 22–28).

The present project has drawn upon the knowledge of recognized TBI biomarker candidates as well as two new candidates; peroxiredoxin 6 (PRDX6) and intercellular adhesion molecule-5/telencephalin (ICAM-5, TLN), for the development of a multivariate analytical approach for the simultaneous assessment of a panel of biomarkers for mTBI (29, 30). This strategy is based upon the proposal that the collective information derived of a multivariate approach mitigates the shortcomings of an individual biomarker that may include limited specificity and/or sensitivity. Each candidate biomarker was selected for its relative brain-specificity and potential to reflect distinct features of TBI mechanisms: (1) neuronal damage and recovery assessed by neuron-specific enolase (NSE) (31–33) and brain derived neurotrophic factor (BDNF) (34, 35); (2) oxidative stress assessed by PRDX6 (30, 36); (3) glial damage and gliosis assessed by glial fibrillary acidic protein (GFAP) and S100 calcium binding protein beta (S100b) (33, 37); (4) immune activation assessed by monocyte chemoattractant protein 1/chemokine (C–C motif) ligand 2 (MCP1/CCL2) (38, 39); and (5) disruption of the intercellular adhesion apparatus assessed by ICAM-5 (40, 41).

The findings presented here demonstrate that fold increases in plasma levels of NSE, PRDX6, S100b, BDNF, and MCP1 biomarkers in adult subjects experiencing mTBI may be formulated to produce an informative TBI assessment score (TBIAS). This TBIAS effectively differentiated between control and mild TBI with improved sensitivity and specificity over that provided by any of the individual biomarkers. It is proposed that the strategy of multivariate analysis can be further developed and refined for both the acute diagnosis of mTBI and the identification of patients who are at risk for developing subsequent clinical complications.

## Materials and Methods

### Clinical samples and institutional review board approvals

Human plasma samples analyzed here were collected as part of two separate clinical studies. The first study, conducted by the National Institute of Neurological Disorders and Stroke (NINDS) of the National Institutes of Health (NIH; Bethesda, MD, USA), investigated suspected TBI in civilians presenting to the emergency department of two Washington DC metropolitan hospitals. The NINDS/NIH/Center for Neuroscience and Regenerative Medicine (CNRM) study was approved by the Central Neuroscience Institutional Review Board (IRB), NIH, the Medstar IRB, Washington DC, USA, and administrative review approval by the Uniformed Services University of the Health Sciences IRB (Bethesda, MD, USA). Following de-identification, the NINDS samples and data were provided through the Center for Neuroscience and Regenerative Medicine Biorepository. A primary objective of the NINDS/NIH study (NCT01132937) was to study the magnetic resonance imaging (MRI) results of individuals who had recently had head injury and suspected traumatic brain injury. Accordingly, blood samples were collected at the times of research neuroimaging: first, upon presentation to the hospital and within 48 h of injury; and second, at a second imaging time ranging between 2 and 7 days post-injury.

The second study, conducted in collaboration with Defence Research and Development Canada, Sunnybrook Health Sciences Centre, St. Michael’s Hospital, and the Department of Surgery and Critical Care Medicine, University of Toronto (all in Toronto, Canada), investigated both healthy volunteers and civilian trauma patients presenting with an isolated moderate-to-severe head injury. The Canadian component of the study was approved by the local REBs of the participating institutions. The biomarker analyses presented here were approved by the Uniformed Services University of the Health Sciences IRB.

This investigation involved two separate sets of control samples. The control samples for the study of mild–moderate TBI consisted of 30 normal adult, age and gender matched, healthy volunteers obtained commercially from Innovative Research (Novi, MI, USA). Control samples for the moderate–severe TBI study consisted of 44 age, gender and race-matched healthy control samples that were collected onsite at the same treatment center where the TBI samples were collected.

### Biomarker analyses

A multiplex assay platform developed for the simultaneous analysis of BDNF, S100b, NSE, GFAP, MCP-1/CCL2, and ICAM-5 in samples of human plasma was used. Plasma levels of PRDX6 were measured using a single-plex assay system.

### TBI 6-plex assay platform

A multiplex analytical platform for six candidate TBI biomarkers (TBI 6-Plex) was established on SECTOR^®^ Imager 6000 reader plates [Meso Scale Discovery (MSD)], Gaithersburg, MD, USA) using the commercially available antibodies and calibrant proteins listed in Table 1.

**Table 1:** **TBI 6-Plex assay reagents**.

Reagent		BDNF	GFAP	MCP1/CCL2	ICAM-5	NSE	S100b
Capture antibody	Vendor	R&D	Genway	R&D	R&D	R&D	Sigma
	Catalog #	MAB848	20-272-190050	MAB679	MAB1950	MAB5169	S2532
Protein calibrant	Vendor	R&D	Calbiochem (EMD)	R&D	R&D	Abnova	Sigma
	Catalog #	2488D005	345996	279-MD-010	1950-M5	H00002026-P01	S6677
Detection antibody	Vendor	R&D	DAKO	R&D	R&D	R&D	Genway
	Catalog #	MAB648	Z0334	AF-279-NA	AF1950	AF5169	18-272-198528

*R&D: R&D Systems Inc., Minneapolis, MN, USA*.

*Sigma: Sigma Aldrich, St Louis, MO, USA*.

*Genway: GenWay Biotech Inc., San Diego, CA, USA*.

*Calbiochem (EMD): Calbiochem, San Diego, CA, USA*.

*Abnova: Abnova, Walnut, CA, USA*.

*DAKO: DAKO, Carpinteria, CA, USA*.

Capture antibodies were printed [20 μg/mL in phosphate buffered saline (PBS)] at addressable locations in the wells of standard-bind multiplex assay plates (MSD proprietary printing service). All detection antibodies were derivatized with electrochemiluminescent SULPHO-Tag NHS-ester R91AN-1 using SULPHO-Tag reagent and procedures provided by MSD. Stock solutions of protein standards (30×) and SULPHO-Tag detection antibodies (10×, 60 μg/mL) were prepared in PBS-1% bovine serum albumin (BSA) and stored at −80°C as single use aliquots. The standard protocol for performing the multiplex assay was as follows. Plates were hydrated for 60 min at room temperature with 25 μL PBS-1% BSA containing immunoglobulin blockers [goat IgG 0.1% (Kerrville, TX, USA)], mouse IgG 0.02% and rabbit IgG 0.1% (Rockland, Gilbertsville, PA). Samples were diluted in an equal volume of PBS-1% BSA and added directly into the wells in a volume of 25 μL. Standard curves were similarly prepared in buffer containing chicken plasma (1:1 dilution in PBS-1% BSA; Innovative Research, Inc.), which served as an off-species matrix to control for the non-specific effects of human plasma. In comparison to other matrices tested (cow, donkey, goat, guinea pig, horse, pig, rabbit; Innovative Research, Inc.), chicken plasma exhibited a low background signal regardless of the volume of matrix assayed, and resulted in standard curves that matched those of human sample containing equal amounts of calibrant. The effects of matrix on the performance of each assay are presented in Table 2. Matrix minimally altered the sensitivity of each assay without affecting its dynamic range.

**Table 2:** **Effects of sample matrix on the lower limit of quantitation and detection (LOQ/LOD) of TBI biomarkers measured by the TBI 6-Plex platform**.

Analyte	Buffer only	Plasma matrix
	LOQ/LOD	LOQ/LOD
BDNF	0.17/0.08	0.06/0.03
GFAP	0.26/0.20	0.27/0.21
NSE	0.24/0.07	0.87/0.28
S100b	0.06/0.02	0.39/0.13
ICAM-5	0.21/0.12	0.51/0.42
MCP1/CCL2	0.003/0.002	0.015/0.015

*LOQ is defined as the biomarker concentration represented by the mean signal for zero concentration plus 10 times the SD of this mean*.

*LOD is defined as the biomarker concentration represented by the mean signal for zero concentration plus three times the SD of this mean*.

*Values = ng/mL*.

*n = 6 replicates per standard curve concentration*.

*Data are representative of three replicate experiments*.

Plates were shaken at room temperature for 2 h and then incubated at 4°C overnight. Wells were washed three times with PBS containing 0.05% Tween-20 (PBS-T) and then incubated for 1 h with a blended mixture of SULPHO-Tagged detection antibodies diluted to 2 μg/mL each in PBS-1% BSA (25 μL/well). The plates were then washed three times with PBS-1% BSA, developed with the addition of MSD Read Buffer (150 μL/well) and read in a SECTOR Imager 6000 Reader (MSD). Lower limits of detection (LOD) and lower limits of quantitation (LOQ) were defined for each assay as 3 times and 10 times the SD of the averaged 0 for each assay, respectively (*n* = 10 for three replicate experiments). Similarly, intra- and inter-assay variability was <3 and 10% respectively, for all assays. All control values for S100b were detected within the range between the LOD and LOQ for the assay, with some rising to the LOQ. Plasma levels of S100b in all post-injury samples were well above the LOQ for the assay. Accordingly, the LOD value was assigned to control samples that did not reach the LOQ.

### PRDX6 single-plex assay platform

The single-plex immunosorbent assay was developed using MSD standard-bind electrochemiluminescence microtiter plates. Combinations of seven monoclonal and five polyclonal anti-PRDX6 antibodies were screened for compatible pairs. A mouse anti-PRDX6 monoclonal antibody (Clone 1A11, GenWay Biotech, catalog #20-007-280008, San Diego, CA, USA) and a rabbit monoclonal anti-PRDX6 antibody (Clone EPR3755, Epitomics, catalog # 2769-1, Burlingame, CA, USA) were optimized as capture and primary antibodies, respectively. The monoclonal capture antibody was purified by Protein G affinity chromatography (Protein G HP SpinTrap, GE Healthcare, Uppsala, Sweden, vendors protocol) to remove glycerol, and then coated to the plates at a concentration of 4 μg/mL in PBS (25 μL/well, overnight, 4°C). The plates were blocked with 3% BSA in PBS for 2 h at room temperature. The wells were then washed three times with PBS containing 0.05% PBS-T and the plasma samples (diluted 1:4 in PBS-1% BSA) were added to wells in a total volume of 100 μL/well. The standard curves were established using serial dilutions of PRDX6 recombinant human protein (GenWay Biotech, catalog #GWB-F65D50, San Diego, CA, USA) in a final volume of 100 μL/well. Standard curves were prepared in PBS-1% BSA containing 25% chicken plasma (Innovative Research, Inc.) to control for the non-specific effects of serum or plasma matrix. Plates were incubated for 2 h at room temperature and washed three times in PBS-T. Following the addition of primary antibody (2 μg/mL in PBS-1% BSA, 25 μL/well), plates were incubated for 1 h at room temperature and washed three times in PBS-T. The plates were then treated with goat anti-rabbit MSD SULFO-Tag antibody (MSD, R32AB-1) (2 μg/mL in PBS-1% BSA, 25 μL/well, and 60 min at room temperature). After three PBS-T washes, the plates were developed with 150 μL/well of MSD Read Buffer T with Surfactant (MSD) and electrochemiluminescence read using a SECTOR Imager 6000 (MSD). The assay can detect 0.7 ng/mL (lower limit of detection; LOD) and is quantitative to 1 ng/mL (lower limit of quantitation, LOQ) as defined by 3 and 10 times the SD of readings obtained for 0 pg/mL standard, respectively (*n* = 10). Values for LOD and LOQ were marginally lower (50%) in the absence of matrix (buffer only). All sample values fell within the linear range of the standard curve. Intra- and inter-assay variability were 4 and 13%, respectively (*n* = 10).

### Statistical analyses

Multivariate analysis of variance (MANOVA) was conducted on plasma levels of seven proteins for each clinical study separately. Violations of normality and homogeneity were addressed using log transformations and adjustments were made to the model to account for difference in sample size between controls and TBI subjects. Additional analyses were conducted to determine differences based on demographics using MANOVA.

The TBIAS was derived first by calculating fold-changes based on the control values and then adding those fold-changes for each marker. Additional MANOVAs were conducted to test the TBIAS model. Ability of the TBIAS to properly classify TBI versus control cases was assessed using receiver operating characteristic (ROC) curves and area under the curve (AUC) analyses for each time point.

## Results

### Patient demographics clinical study investigating mild to moderate TBI

The NINDS/NIH/CNRM study enrolled 154 subjects with head trauma: direct head impact 54% (83 patients), falls 28% (43 patients), acceleration/deceleration injuries (mainly automobile accidents) 12% (18 patients), and other 7% (10 patients). Inclusion and exclusion criteria for this study are presented in Table S1 in Supplementary Material. The median Glasgow coma scale score at the time of admission was 15. Clinical data also included information concerning loss of consciousness, post-traumatic amnesia, computed tomography (CT) scan, and MRI results. An analysis of the available CT and MRI data revealed that 51% (79 patients) of the TBI subjects presented with no discernable CT or MRI abnormalities, whereas 23% (36 patients) presented with CT and MRI abnormalities and 16% (24 patients) with MRI imaging abnormalities. No imaging data were available on 10% (15 patients). The cohort of subjects with imaging findings related to moderate TBI had a median Glasgow coma scale score of 14 and periods of loss of consciousness and post-traumatic amnesia intervals consistent with mild TBI (<30 min and ≤24 h, respectively). However, based upon the accepted guidelines for clinical classification, the subset of image-positive subjects was classified here as having moderate TBI (*n* = 60), and the remaining image-negative subjects (*n* = 79) were classified as having experienced a mild TBI (42). Patient demographics and clinical data are presented in detail in Table S2 in Supplementary Material. Control samples for comparison were obtained commercially from Innovative Research (Novi, MI, USA).

### Patient demographics for the clinical study investigating moderate to severe TBI

This study involved a total of 106 subjects who were admitted with isolated head injuries and diagnosed with moderate to severe TBI. Inclusion and exclusion criteria for this study are presented in Table S1 in Supplementary Material. Plasma samples were obtained at admission into the emergency department and at 6, 12, and 24 h post-injury. Glasgow coma scale scores on admission were all below 12 (range = 3–12; mean = 6; median = 6) and 26 of 106 subjects died within 24 h following admission. A total of 44 control samples were collected as part of this study from non-trauma, non-post-traumatic stress disorder military personnel. The demographic and clinical data available from this study were limited as compared to the data recorded in the mild to moderate TBI study. Patient demographics and clinical data are presented in detail in Table S3 in Supplementary Material.

### Effects of mild to moderate TBI on plasma levels of candidate biomarker proteins

Of the 154 subjects, 45 did not have imaging and/or plasma samples at all-time points. Therefore, they were not included in the initial analyses. Table 3 presents the mean plasma levels of all 7 biomarker for the remaining 109 TBI subjects. Statistical comparisons between control and TBI data were made using a MANOVA. Importantly, plasma biomarker responses were not different in subjects who had positive neuroimaging findings (*n* = 43) versus those who did not [*n* = 79; *F*(6, 128) = 0.0658, *p* > 0.05]. Accordingly, all TBI subjects were combined together for further analyses. The overall multivariate model was significant for each time point [TP1: *F*(6, 128) = 47.33, *p* < 0.001; TP2: *F*(7, 111) = 40.09, *p* < 0.001] with effect size >0.14 and observed power of 1.0. The statistical data presented in Table 3 indicate that blood levels of BDNF, MCP1/CCL2, NSE, S100b, and PRDX6 were significantly greater in TBI subjects as compared to controls. Blood levels of GFAP were consistently below the level of quantification for all subjects and all-time points. Finally, there were no differences in circulating levels of ICAM-5 between the control and TBI subjects.

**Table 3:** **Plasma levels of candidate TBI biomarker proteins in subjects with mild to moderate TBI and controls**.

Condition	ng/mL	BDNF	ICAM-5	MCP1/CCL2	NSE	S100b	PRDX6	GFAP
Control	Mean	2.8	0.9	0.1	30.0	0.2	78.2	<0.3
	SEM	0.6	0.1	0.0	6.2	0.0	17.5	–
Admission	Mean	9.1	0.9	0.2	55.5	0.7	388.0	<0.3
	SEM	0.6	0.0	0.0	2.8	0.1	15.0	–
	*p* =	<0.0001	NS	<0.001	<0.0001	<0.03	<0.0001	–
2–7 days	Mean	9.4	0.9	0.2	67.3	0.6	430.8	<0.3
	SEM	0.6	0.0	0.0	3.3	0.1	15.8	–
	*p* =	<0.0001	NS	<0.001	<0.0001	<0.03	<0.0001	–

The effects and interactions among demographic variables and neurological findings on plasma biomarker responses were evaluated by MANOVA. As noted above for neuroimaging, when co-varying for gender, race, age, and education, there was no overall difference between males and females and no differences with regard to race and education. Younger subjects (<45 years) had modestly lower levels of MCP1/CCL2 at admission as compared to levels measured in older (>45 years) individuals (*p* > 0.05). No other biomarkers showed this pattern. Changes in the levels of plasma biomarkers did not reflect the nominal differences observed in admission Glasgow coma scale or CT/MRI imaging results.

**Figure 1:**
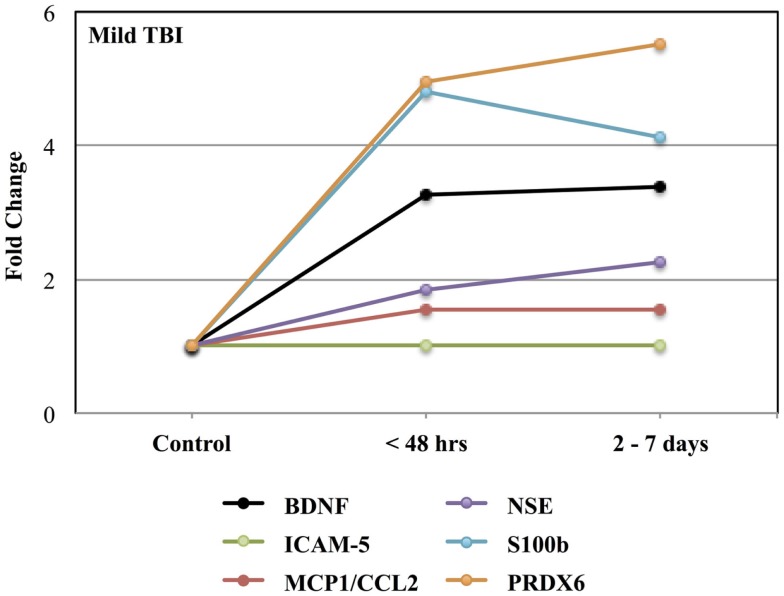
**Effects of mild to moderate TBI on plasma levels of candidate biomarker protein expressed as fold-changes from controls values**. See Table 3 for statistical significance (*p* values).

Figure 1 shows the time-related fold increases in mean plasma levels observed for BDNF, ICAM 5, MCP1/CCL2, NSE, S100b, and PRDX6 following mild to moderate TBI.

### Effects of moderate to severe TBI on plasma levels of candidate biomarker proteins

Table 4 presents the mean plasma levels for all biomarkers at all-time points for TBI subjects and control subjects comprising the moderate to severe study. Compared to control values, circulating levels of all biomarkers were increased in response to moderate to severe TBI. While the mean levels of GFAP in the TBI group rose into the detectable range following moderate to severe TBI, the statistical significance of this response could not be calculated because control values were below the limit of detection. Blood levels of all other markers were significantly increased following TBI.

**Table 4:** **Plasma levels of candidate TBI biomarker proteins in subjects with moderate to severe TBI**.

Condition	ng/mL	BDNF	ICAM-5	MCP1/CCL2	NSE	S100b	PRDX6
Control	Mean	1.2	1.4	0.1	10.5	0.3	144.5
	SEM	0.2	0.1	0.0	0.9	0.0	24.2
Admission	Mean	4.5	1.1	0.4	120.0	2.5	762.9
	SEM	0.5	0.1	0.1	10.0	0.6	85.5
	*p* =	<0.001	<0.08	<0.001	<0.001	<0.001	<0.001
6 h	Mean	4.0	1.0	0.3	110.8	0.6	510.5
	SEM	0.4	0.1	0.1	9.3	0.1	53.2
	*p* =	<0.001	<0.001	<0.001	<0.001	<0.002	<0.001
12 h	Mean	3.3	1.0	0.2	100.4	0.6	413.2
	SEM	0.4	0.1	0.0	9.3	0.1	43.5
	*p* =	<0.001	<0.001	<0.001	<0.001	<0.01	<0.001
24 h	Mean	2.3	1.0	0.3	69.2	0.6	280.0
	SEM	0.3	0.1	0.0	7.4	0.2	44.4
	*p* =	<0.001	<0.001	<0.005	<0.001	<0.07	<0.006

**Figure 2:**
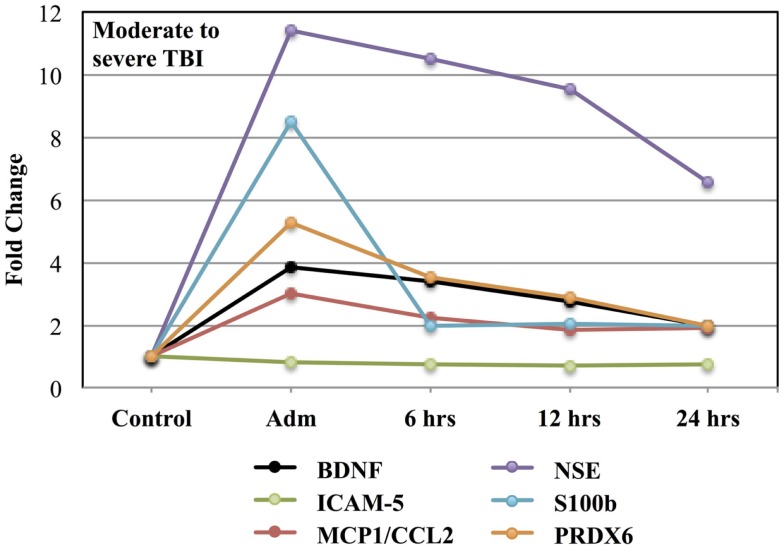
**Effects of moderate to severe TBI on plasma levels of candidate biomarker proteins expressed as fold-changes from control values**.

Figure 2 shows the time course for the fold-changes in plasma levels of candidate biomarker proteins following moderate to severe TBI. Time-related increases (*p* < 0.05) in plasma levels were observed for five of the seven candidate biomarker proteins: BDNF, MCP1/CCL2, NSE, S100b, and PRDX6, whereas, circulating levels of ICAM-5 were not altered. A fold-change in blood levels of GFAP could not be calculated because control values were below the limit of detection.

### Formulation of a TBI assessment score

The distinctive profiles observed in blood levels of biomarker proteins following TBI suggested that these patterns could contribute to formulation of a biomarker signature for the diagnosis of TBI. Tables 5 and 6 present fold-changes over control values in biomarkers proteins observed at the time of admission for the mild to moderate and moderate to severe TBI studies, respectively. Table 7 shows the formulation of a TBIAS, which is based upon the summation of the fold-changes observed in plasma levels of BDNF, MCP1/CCL2, NSE, S100b, and PRDX6. Because control values are fixed by condition, each of biomarkers was assigned a value of one, for a summation control score of five. In the case of mTBI, a summation score of 17 was calculated for the composite fold-changes in candidate biomarkers. In the case of moderate to severe TBI, the summation score was 32. Calculated in this fashion, the TBIAS distinguishes mTBI from controls and from moderate to severe TBI.

**Table 5:** **Mean plasma values and respective fold-change in values of candidate TBI biomarker proteins in subjects with mild to moderate TBI**.

	BDNF	ICAM-5	MCP1/CCL2	NSE	S100b	PRDX6	GFAP^a^
Control	2.8 ± 0.6	0.9 ± 0.1	0.1 ± 0.0	30.0 ± 6.2	0.2 ± 0.0	78.0 ± 17.5	<0.3
TBI admission	9.1 ± 0.6	0.9 ± 0.0	0.2 ± 0.0	55.5 ± 2.8	0.7 ± 0.1	388.0 ± 15.0	<0.3
Fold-change	3	No Δ	2	2	5	5	–

*^a^No fold-change could be calculated for GFAP because all values in both control and TBI groups were below the level of quantification*.

**Table 6:** **Mean plasma values of candidate TBI biomarker proteins in moderate to severe TBI subjects and their respective fold-changes compared to controls**.

	BDNF	ICAM-5	MCP1/CCL2	NSE	S100b	PRDX6	GFAP^a^
Control	1.2 ± 0.2	1.4 ± 0.1	0.1 ± 0.0	10.0 ± 0.9	0.3 ± 0.0	145.0 ± 24.0	<0.3
TBI admission	4.5 ± 0.5	1.1 ± 0.1	0.4 ± 0.1	120.0 ± 10.0	2.5 ± 0.6	763.0 ± 85.0	3.8 ± 1.5
Fold-change	4	No Δ	3	11	9	5	–

*^a^No fold-change could be calculated for GFAP because control values were below the level of quantification*.

**Table 7:** **Formulation of a TBI assessment score**.

Protein	Control	Mild to moderate	Moderate to severe
BDNF	1	3	4
MCP1/CCL2	1	2	3
NSE	1	2	11
S100b	1	5	9
PRDX6	1	5	5
TBI score	5	17	32

**Figure 3:**
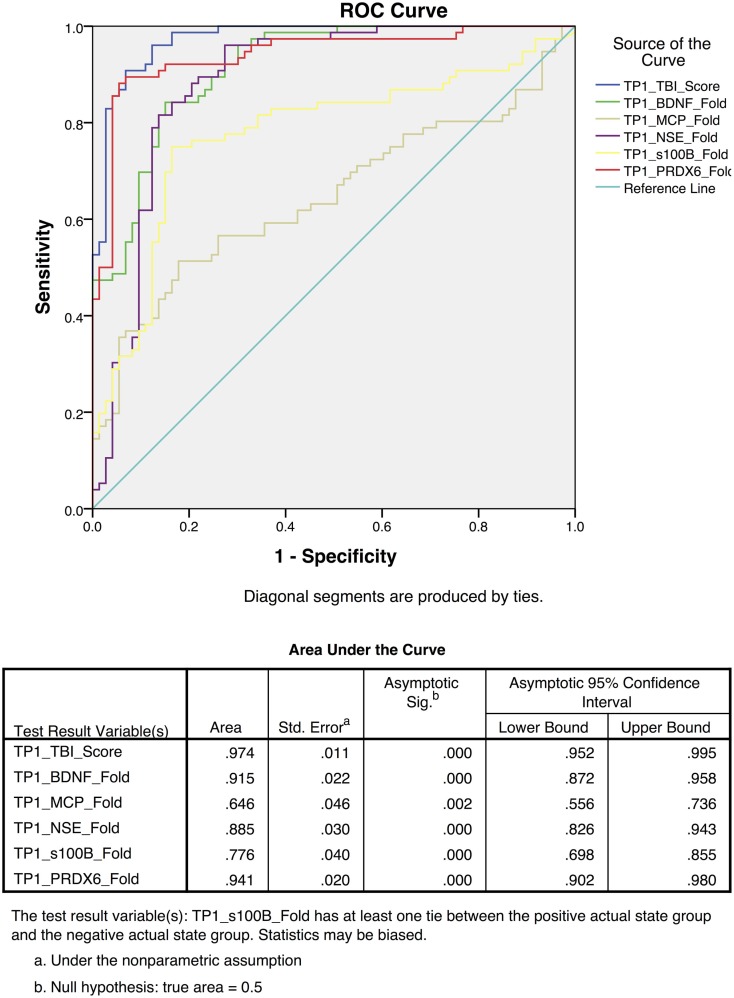
**Individual receiver operating characteristic curves for time point 1 (TP1) TBI assessment scores from the mild to moderate cohort**. The red line demonstrates the combined TBI assessment score ROC curves for five TBI Biomarkers. Note the high sensitivity and specificity [AUC 0.97 95% CI (0.95–0.99)] when comparing individual biomarkers as a collective.

**Figure 4:**
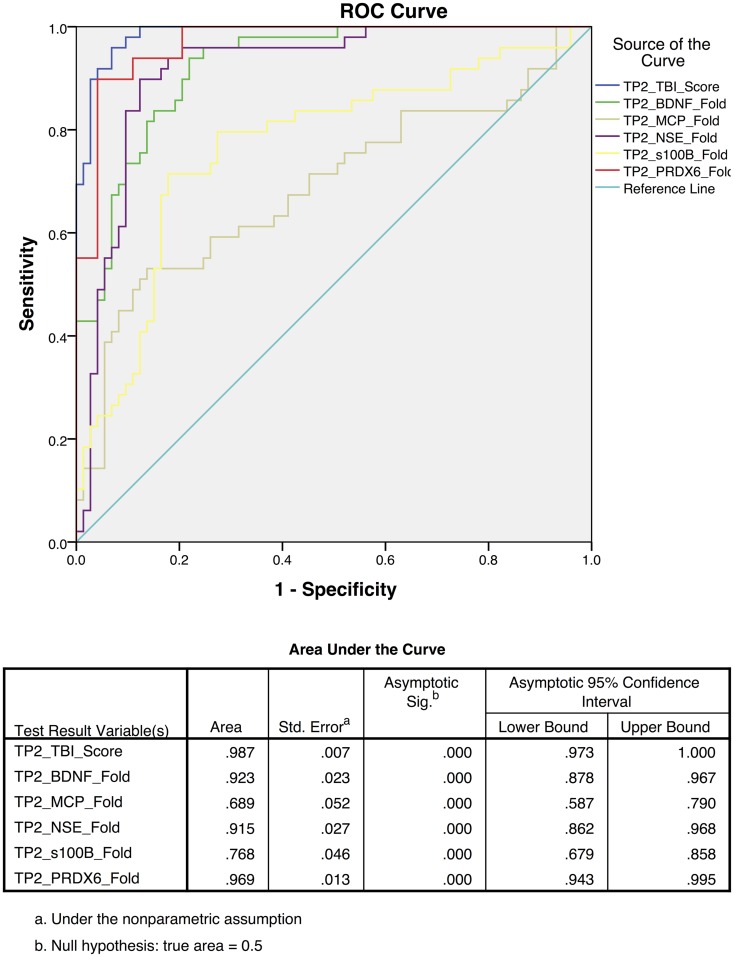
**Receiver operating characteristic (ROC) curves for the assessment scores for the mild to moderate TBI cohort at 2–7 days post-injury [time point 2 (TP2)]**. The blue line represents the combined TBI assessment score ROC curve (TP2_TBI_Score), for data derived from all five biomarker candidates. Highest sensitivity and specificity [AUC 0.987 95% CI (0.97–1.0)] were observed when considering all five biomarkers collectively.

To differentiate TBI from control subjects, receiver operating characteristic (ROC) curves were calculated for time point 1 (TP1; ED admission; Figure 3) and time point 2 (2–7 days post-injury; Figure 4). Accuracy of such a test is reflected by the AUC where an area of 1.0 represents a perfect test and an AUC above 0.9 is considered excellent. The AUC for the TBIAS at time point one was 0.974 (SE.011) while the next highest biomarker was PRDX6 (0.935/0.019). At time point two (TP2) 2–7 days post-injury, the AUC for the TBIAS was 0.987 (0.007) while the next highest biomarker was again PRDX6 (0.967/0.012). To test the accuracy of the TBIAS for mTBI, subjects with positive imaging results were removed from the data and ROC curves were recalculated. There was no change in the AUC.

## Discussion

Despite significant scientific effort investigating the mechanisms and neuropathology of TBI, no biomarkers have been established for diagnosing milder forms of TBI, assessing its underlying mechanisms, identifying effective therapeutic targets, or predicting future risks (13, 21, 43, 44). In this regard, it has been proposed that a biomarker signature based upon multiple biomarker candidates can improve the diagnosis and monitoring of mTBI (13, 28). The present investigation demonstrates that five of the biomarkers studied here can be used to establish a signature that identifies mTBI with a quantitative assessment score. Because the assessment score is based upon definitive measures of circulating biomarkers, it provides an objective assessment that is easily standardized across clinical settings.

The present study demonstrates how a profile of biomarker responses can be combined in a simplistic manner to create an informative assessment score that identifies mTBI. This assessment score is based upon fold-changes in plasma levels of both recognized and novel biomarker proteins measured in humans experiencing mild to moderate TBI. It is generally held that when considered individually, none of the proteins involved have proven universally informative in the diagnosis of milder forms of TBI (28). The present data suggest that a multivariate analysis may result in a diagnostic tool having improved clinical utility for mild TBI. While the TBIAS distinguishes between the milder versus severe forms of TBI, the most important use for this type of analysis will be in the assessment of mTBI where definitive imaging data cannot be obtained.

The multivariate approach used here evaluates proteins selected for their potential to detect injury and classify clinical outcomes in an acute, subclinical phase. Important strengths of multiplex analysis used here include conservation of precious samples and the ability to simultaneously evaluate, under identical assay conditions, a panel of analytes that can together provide insights into a variety of components of an injury, which in this case include neuronal injury [NSE, ICAM-5, BDNF; (45, 46)], glial activation and injury [GFAP, S100b; (47, 48)], oxidative stress [PRDX6; (49, 50)], and neuroinflammation [MCP1/CCL2; (51)].

Consistent with other studies, the present findings indicate that blood levels of TBI biomarkers generally increase very quickly to a maximum within 24 h post-injury (52–55). After this period, however, the profile of biomarker proteins in blood varies over time, presumably reflecting different rates of production and clearance for each protein (Figures 1 and 2). While the acute post-injury sample provides a relatively standardized time point for clinical comparisons, samples collected at later times post-injury may prove informative in the assessment of patient-specific pathogenic processes (56, 57).

This study has several limitations. First, the study has a modest sample size and will need to be validated in a larger independent cohort. Second, the study design focused on the evaluation of acute TBI and did not determine the relations that may exist between an acute biomarker signature and the development of longer-term complications or increased susceptibility to a subsequent mTBI resulting in a second injury syndrome. Third, given the unexpected nature of TBI, pre-injury blood samples were not available. Accordingly, baseline measures were derived from the analysis of standardized control samples. While this approach provides for an important comparison across studies, within-subject responses are unavailable for individualized diagnoses. Fourth, due to the acute nature of the study designs, it was not possible to identify those at particular risk for developing neuropathologies as a consequence of a mTBI event. Lastly, this investigation is limited by the selection of the candidate biomarkers for study. The present panel of proteins can certainly be improved upon with the inclusion of additional biomarker candidates; both recognized and newly discovered, which together may offer greater diagnostic sensitivity and specificity as well as important clinical insight into underlying mechanisms and potential long-term consequences of a mTBI.

While not often emphasized, it should be recognized that many, if not all of the candidate TBI biomarkers currently under investigation are not strictly brain-specific. For example, GFAP is expressed in the gastrointestinal tract and the liver (58), BDNF and NSE are concentrated in platelets (59, 60) and S100b is expressed in adipocytes and the GI tract (61, 62). Similarly, ubiquitin carboxy terminal hydrolase-L1 (UCH-L1, PGP9.5), an emerging prototype TBI biomarker, is expressed in the endometrium and secretory cells of the pituitary gland (63–66). Accordingly, the concept of a brain-specific response must be qualified when considering TBI biomarker proteins and should include the potential contribution of poly trauma to a biomarker signature. In this regard, an additional strength in a panel of TBI biomarkers is that it can provide a multi-dimensional view of a brain injury, avoiding possible confounding variables that may result from poly trauma and the assessment of a single biomarker (67).

**Figure 5:**
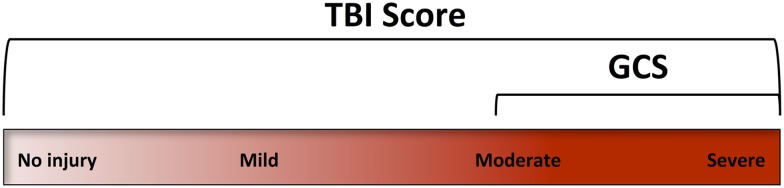
**The Glasgow coma scale (GCS) was designed to assess levels of consciousness**. Accordingly, its clinical use focuses on the more severe forms of TBI. A TBI assessment score (TBIAS) may be used to assess the entire spectrum of TBI providing information on specific pathogenic mechanisms that may be unique to an individual’s injury.

In conclusion, this research demonstrates that a profile of blood biomarker responses can be used to formulate a novel diagnostic score “TBIAS” that is sensitive for the detection of mTBI. Ideally, this multivariate TBIAS will be refined with the discovery of additional sensitive and specific biomarkers that can assess the entire spectrum of a TBI including physical injury, metabolic dysfunction, oxidative stress, neuroinflammation, and changes in cerebrovascular regulation (Figure 5). Ideally, a multivariate biomarker signature will also be able to identify those at particular risk for developing serious neuropathologies as consequence of a subsequent mTBI event. Overall, the TBIAS strategy discussed here has potential for clinical decision-making and for monitoring the progression of injury and/or recovery. As such, a TBIAS could have a role in managing patients at high-risk of repeated injury and may be incorporated into guidelines for return to work, duty, or play.

## Author Contributions

All authors were involved in the drafting the manuscript or revising it critically for important intellectual content to include the final approval of this version for publication. JB, AY, RL, MM, RA, and GM substantially contributed to the conception and design of the work, analysis and interpretation of data. LL, SR, AB, and SR substantially contributed to the acquisition of data. All agree to be accountable for all aspects of the work in ensuring that questions related to the accuracy or integrity of any part of the work are appropriately investigated and resolved.

## Conflict of Interest Statement

The authors declare that the research was conducted in the absence of any commercial or financial relationships that could be construed as a potential conflict of interest. The Associate editor Angela M. Boutte declares that, despite being affiliated to the same institution as author Angela M. Yarnell, the review process was handled objectively.

## Supplementary Material

The Supplementary Material for this article can be found online at http://journal.frontiersin.org/article/10.3389/fneur.2015.00068
