# Supplementary material for: Multivariate Analysis of Traumatic Brain Injury: Development of an Assessment Score
Source: Front Neurol. 2015 Mar 30;6:68. doi: 10.3389/fneur.2015.00068 (PMC4378282; doi:10.3389/fneur.2015.00068)
Supplement: Supplementary file 1 [file datasheet_1.docx]

***Supplementary Material***

**Multivariate analysis of traumatic brain injury: development of an assessment score**

**John E. Buonora^1,2^, Angela M. Yarnell^3^, Rachel C. Lazarus^1^, Michael Mousseau^1^, Lawrence L. Latour^4,7^, Sandro B. Rizoli^5^, Andrew J. Baker^6^, Shawn G. Rhind^7^, Ramon Diaz-Arrastia^8^, Gregory P. Mueller^1*^**

^1^Department of Anatomy, Physiology and Genetics, Uniformed Services University of the Health Sciences, Bethesda, Maryland, USA.

^2^U.S. Army Graduate Program in Anesthesia Nursing, Academy of Health Sciences, Joint Base San Antonio, Fort Sam Houston, TX, USA.

^3^Behavioral Biology Branch, Center for Military Psychiatry & Neuroscience Research, Walter Reed Army Institute of Research, Silver Spring, MD, USA.

^4^Stroke Branch, National Institute of Neurological Disorders and Stroke, Bethesda, Maryland, USA.

^5^Keenan Research Centre of the Li Ka Shing Knowledge Institute, St Michael's Hospital, Departments of Anesthesia, Surgery & Critical Care Medicine, University of Toronto, Toronto, Ontario, Canada.

^6^Brain Injury Laboratory, Li Ka Shing Knowledge Institute, Cara Phelan Centre for Trauma Research Keenan Research Centre University of Toronto, Toronto, Ontario, Canada.

^7^Defence Research & Development Canada, Toronto Research Centre, Toronto, Ontario, Canada.

^8^Center for Neuroscience and Regenerative Medicine, Uniformed Services University of the Health Sciences, Bethesda, Maryland, USA.

*** Correspondence:** Gregory P. Mueller, Ph.D., Uniformed Services University of the Health Sciences (USU), Department of Anatomy, Physiology and Genetics, Room C2117, 4301 Jones Bridge Road, Bethesda, Maryland 20814-4799, gregory.mueller@usuhs.edu

1. **Supplementary Data**

N/A

1. **Supplementary Figures and Tables**

## Supplementary Tables

## Table S1. Inclusion and Exclusion Criteria

**Mild to Moderate TBI Study** (<https://clinicaltrials.gov/ct2/show/NCT01132937>):

Inclusion:

- History of acute head injury with or suspected non-penetrating acute TBI
- Age 18 years or older
- Deemed medically safe for study participation by the subject’s attending physician
- Able to provide consent or have a legally-authorized representative provide consent.

Exclusion:

- Considered to be psychiatrically unstable by the patient’s attending physician
- Contraindication to MRI scanning including: pacemakers or other implanted electrical devices, brain stimulators, some types of dental implants, aneurysm clips (metal clips on the wall of a large artery), metallic prostheses (including metal pins and rods, heart valves, and cochlear implants), permanent eyeliner, implanted delivery pump, or shrapnel fragments.
- Conditions precluding entry into the scanner such as morbid obesity or claustrophobia or conditions requiring sedation.
- In female subjects, pregnancy.

**Moderate to Severe TBI Study:**

Inclusion:

- TBI Patients with isolated moderate to severe head trauma defined as a Glasgow Coma Scale (GCS) score < 9
- Non-head Abbreviated Injury Score (AIS) < 2 and moderate (GCS > 9) TBI
- Admitted to Sunnybrook Health Sciences Centre or St. Michael¹s Hospital.

Exclusion:

- Elapsed time between the trauma and admission to the emergency department exceeding 3 hours
- Age less than 16 years
- Pregnancy
- Absence of vital signs prior to admission to the ED.

## Table S2. Demographic characteristics and clinical variables for subjects with mild-moderate TBI.

##

|  |  | **TBI** |  | **Controls** |
| --- | --- | --- | --- | --- |
|  |  | **N = 154** |  | **N = 30** |
| **Age** |  |  |  |  |
|  | Mean, years (SD) | 47 (19) |  | 25 (5) |
|  | Median | 45.8 |  | 24 |
|  | Range | 19-91 |  | 19-50 |
| **Gender (%)** |  |  |  |  |
|  | Male | 103 (67) |  | 15 (50) |
|  | Female | 43 (28) |  | 15 (50) |
|  | Unknown | 8 (5) |  | 0 (0) |
| **Race (%)** |  |  |  |  |
|  | Caucasian | 103 (67) |  | 23 (76) |
|  | Non-Caucasian | 30 (19) |  | 7 (24) |
|  | Unknown | 21 (14) |  | 0 (0) |
| **Education (%)** |  |  |  |  |
|  | < Grade 12 | 4 (3) |  |  |
|  | High School / equivalent /Associates | 70 (45) |  |  |
|  | Bachelor's degree | 27 (17) |  |  |
|  | PhD/Professional | 30 (19) |  |  |
|  | Unknown | 23 (15) |  | 30 (100) |
| **Mechanism of Injury (%)** |  |  |  |  |
|  | Acceleration/deceleration | 18 (12) |  | 0 |
|  | Fall | 43 (28) |  | 0 |
|  | Direct impact | 83 (54) |  | 0 |
|  | Unknown | 10 (7) |  | 0 |
| **Glasgow Coma Scale score in ED (%)** |  |  |  |  |
|  | < 9 | 3 (2) |  | 0 |
|  | 9 - 12 | 3 (2) |  | 0 |
|  | ≥ 13 | 132 (86) |  | 30 (100) |
|  | Unknown | 16 (10) |  | 0 |
| **Loss of Consciousness (%)** |  |  |  |  |
|  | Yes | 69 (45) |  | 0 |
|  | No | 58 (37) |  | 30 (100) |
|  | Unknown | 27 (18) |  | 0 |
| **Post-traumatic amnesia (%)** |  |  |  |  |
|  | Yes | 83 (54) |  | 0 |
|  | No | 67 (43) |  | 30 (100) |
|  | Unknown | 4 (3) |  | 0 |
| **Imaging – CT and MRI** |  |  |  |  |
|  | Positive | 36 (23) |  | 0 |
| **Imaging - MRI** |  |  |  |  |
|  | Positive | 24 (16) |  | 0 |
| **Admitted to Hospital (%)** |  |  |  |  |
|  | Admitted | 100 (65) |  | 0 |
|  | Unknown | 32 (20) |  |  |
| **Neurobehavorial symptom inventory score** |  |  |  |  |
| 22 symptoms scoring 0-4 | Mean | 0.75 |  | N / A |
| **Extended Outcome 30-day Post-injury** |  |  |  |  |
| Satisfaction with life scale (1-35) (n=26) | Mean | 23 |  | N / A |
| Glasgow outcome scale extended (1-8) (n=59) | Mean | 6.5 |  | N / A |
| **Extended Outcome 90-day Post-injury** |  |  |  |  |
| Satisfaction with life scale (1-35) (n=26) | Mean | 24 |  | N / A |
| Glasgow outcome scale extended (1-8) (n=61) | Mean | 7 |  | N / A |

**Table S3. Demographic characteristics and clinical variables for subjects with moderate to severe TBI study.**

|  | | **TBI** |  | **Controls** |
| --- | --- | --- | --- | --- |
|  | | **N = 106** |  | **N = 44** |
| **Age** |  |  |  |  |
|  | Mean, years (SD) | 47 (21) |  |  |
|  | Median | 45.5 |  |  |
|  | Range | 16-96 |  |  |
|  | Unknown | 0 |  | 44 (100) |
| **Gender (%)** |  |  |  |  |
|  | Male | 85 (80) |  |  |
|  | Female | 21 (20) |  |  |
|  | Unknown | 0 |  | 44 (100) |
| **Glasgow Coma Scale score in Emergency Department (%)** |  |  |  |  |
|  | < 9 | 75 (71) |  | 0 |
|  | 9 - 12 | 28 (26) |  | 0 |
|  | ≥ 13 | 0 (0) |  | 44 (100) |
|  | Unknown | 3 (3) |  | 0 |
| **Marshall Score (%)*** |  |  |  |  |
|  | I | 15 (14) |  | 0 |
|  | II | 51 (48) |  | 0 |
|  | III | 9 (8) |  | 0 |
|  | IV | 18 (17) |  | 0 |
|  | V | 9 (8) |  | 0 |
|  | VI | 0 |  | 0 |
|  | Unknown | 4 (4) |  | 0 |
| **Surgery in first 24 hours (%)**** |  |  |  |  |
|  | Yes | 28 (26) |  | 0 |
|  | No | 78 (74) |  | 0 |
|  | Unknown | 0 |  | 0 |
| **Dead (%)** |  |  |  |  |
|  | % | 26 (24) |  | 0 |
| **Glasgow outcome scale extended at Hospital Discharge (1-8) (n=27)***** |  |  |  |  |
|  | Mean | 3.2 |  | 0 |

*A rating scale with 6 categories, used to predict both the risk of increased intra-cranial pressure and outcome in adults (Category 1 = diffuse injury, no visible pathology - Category 6 = major CT abnormality (Marshall et al., 1992).

** Craniotomies and decompressive craniectomies.

*** A rating scale with 8 categories was used to measure outcome and clinical status 6 months after injury. (1 = severe disability and poor outcome; 8 = highly functional and good outcome (Wilson et al., 1998).

**References:**

Marshall LF, Marshall SB, Klauber MR, Van Berkum Clark M, Eisenberg H, Jane JA, et al. The diagnosis of head injury requires a classification based on computed axial tomography. J Neurotrauma (1992) 9(Suppl 1):S287–92.

Wilson JT, Pettigrew LE, Teasdale GM. Structured interviews for the Glasgow outcome scale and the extended Glasgow outcome scale: guidelines for their use. J Neurotrauma (1998) 15:573–85. doi:10.1089/neu.1998.15.573
